# A Lithium Fluoride Interfacial Layer for Low-Voltage and Reliable Perovskite Memristors

**DOI:** 10.1021/acsaelm.5c02347

**Published:** 2025-12-19

**Authors:** Naresh Kumar Pendyala, Ignacio Sanjuán, Qun-Gao Chen, Wen-Ya Lee, Chu-Chen Chueh, Antonio Guerrero

**Affiliations:** † Institute of Advanced Materials (INAM), 16748 Universitat Jaume I, 12006 Castelló, Spain; ‡ Department of Chemical Engineering and Biotechnology, 34877 National Taipei University of Technology, Taipei 106344, Taiwan; § Department of Chemical Engineering, 33561 National Taiwan University, Taipei 10617, Taiwan

**Keywords:** memristor, halide perovskite, buffer-layer, lithium fluoride, low-voltage, cycling stability

## Abstract

Halide-perovskite materials have emerged as promising candidates for constructing reliable memristors, a key element for advancing neuromorphic computing systems. While several perovskite formulations have been tested, the nature of the external interfaces has not been exploited to its full potential. In this study, LiF is employed as an interfacial layer between a bromide-perovskite and the top contact. The interlayer acts as a source of Li^+^ ions that facilitate the formation of conducting filaments, combining the high ionic conductivity of a halide perovskite and the small size of the Li^+^ ion. The incorporation of a LiF layer significantly enhances device performance at low operation voltages (∼70–150 mV) with a gradual increase in conductance, rendering the devices suitable for analog computation. Overall, devices yield stable and highly reproducible results with high sensitivity to the external voltage. Notably, these devices demonstrate high cycling stability during >10^4^ cycles with small variability in writing–erasing measurements. These findings underline the potential of LiF-enhanced memristors for reliable and energy-efficient neuromorphic computing applications. As a proof of concept, these low-voltage memristors successfully functioned as synaptic weights in an emulated deep neural network (DNN) for handwritten digit recognition. Importantly, the use of LiF as an interlayer should be universally valid for other families of materials used in memristor applications.

## Graphical abstract

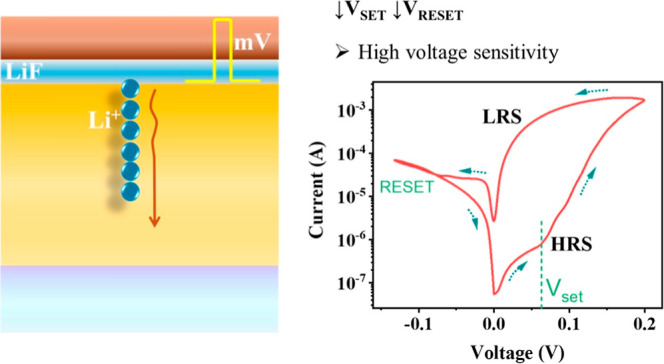

## Introduction

Neuromorphic computing is a highly attractive field of research that mimics the human brain for the upcoming low-energy in-memory functional technology demands.^1–4^ The development of neuromorphic systems with analog switching and multilevel states is of paramount importance and can offer a significant leap toward low-energy, high-efficiency computing. To date, most research has focused on metal oxide memristors, i.e., TiO_2_ or HfO_2_, which support nanoscale fabrication and heavily rely on oxygen vacancies to facilitate the conductive transitions.^5,6^ However, their practical applicability hinges on simultaneously meeting several critical criteria, including stability, reproducibility, and, most importantly, the energy consumption for each memory event.^7–10^

Alternatively, halide perovskites are emerging as a highly attractive material for memristor fabrication due to their solution processability and unique combination of ionic and electronic conductivity.^11–13^ In general, halide perovskite memristors are found to be very efficient with a large hysteresis when interlaid with a thin buffer layer between the perovskite and the contacts.^14^ These buffer layers act as a protective barrier against environmental degradation. At the same time, the buffer layers avoid the direct contact between the perovskite and the metal contact, thereby controlling the charge accumulation/transfer at the interface and also the ion migration from the metal layer to the perovskite.^15^ Thus, the switching behavior of the device can be precisely controlled, depending on the properties of the buffer layer. Several buffer layer materials have been incorporated into the perovskite memristors over the past few years, including poly­(methyl methacrylate) (PMMA), poly­(3,4-ethylenedioxythiophene) polystyrenesulfonate (PEDOT/PSS), or graphene.^16–18^ On the other hand, metal oxides such as titanium oxide (TiO_2_), aluminum oxide (Al_2_O_3_), or zinc oxide (ZnO) have also been used as interfacial layers. Since metal oxides can be used themselves as active layers in memristors, there is a fair question about the role of these interfaces. In general, the role of the interfacial layer in perovskite memristors is an area that still needs to be understood to further improve the performance of the devices.

Recently, we have systematically investigated a series of metal and metal halide interfacial layers for halide perovskite-based memristors.^19^ Our study included nearly inert metals (Au and Pt), low-reactive contacts (Cu), high-reactive contacts (Ag and Al), and preoxidized metals. It was found that the use of preoxidized silver in the form of AgI served as a very promising interfacial material that can be activated at a relatively low voltage (∼0.2 V) during cyclic voltammetry measurements, promoting Ag filamentary formation as the most relevant switching mechanism with high reproducibility. Importantly, our work highlighted the critical role of the buffer layer in achieving a low activation applied voltage in halide perovskite memristors by using a preoxidized metal prone to migration under the presence of an electrical field. Particularly, when we explored AgI with MAPbBr_3_, it exhibited significantly enhanced stability and reliability during electrical measurements.^20^

Although AgI has proven effective in facilitating device switching from a high-resistive state (HRS) to a low-resistive state (LRS) at reduced *V*_set_ values, the quest for materials with smaller metal ions remains essential to achieve lower energy devices. The presence of smaller ions in the interfacial layer can substantially enhance ionic mobility and more efficiently promote the formation and dissolution of conductive filaments at lower potentials. Such materials have the potential to further improve switching performance, reduce energy consumption, and increase the speed, responsiveness, and overall reliability.

In this work, we therefore investigated the use of lithium fluoride (LiF) as a buffer layer as a source of Li^+^ ions that can easily migrate through the halide perovskite matrix. Memristor devices showed reliable operation at low voltages, with endurance of more than 10,000 cycles using low voltages for the writing process during a train of pulses. As a proof of concept, the performance of the device was tested using a deep neural network (DNN) for pattern recognition tasks. The halide perovskite devices containing LiF achieved acceptable results in neuromorphic computing applications driven by low operating voltages.

## Results and Discussion

The device is fabricated on a glass|FTO conducting substrate, onto which an organic buffer layer, poly­(3,4-ethylenedioxythiophene) polystyrenesulfonate (PEDOT/PSS), is deposited. This buffer layer plays a crucial role in enhancing the wettability of the perovskite precursor and mitigating current leakages, ensuring an efficient device performance. The semiconducting layer of the device is composed of methylammonium lead bromide (MAPbBr_3_), a perovskite material chosen for its excellent optoelectronic properties and better stability as compared to other halide perovskites. A thin LiF interfacial layer is deposited as a source of Li^+^ ions, followed by evaporation of a top Ag electrode to optimize the charge collection. Figure 1a shows the cross-sectional scanning electron microscopy (SEM) image of the complete device structure (glass|FTO|PEDOT/PSS|MAPbBr_3_|LiF|Ag). This SEM image demonstrates that all layers have a uniform thickness and a highly crystalline MAPbBr_3_ layer of ∼400 nm. The inorganic LiF interfacial layer is conformal to the perovskite layer with a thickness of about 30 nm.

**Figure 1:**
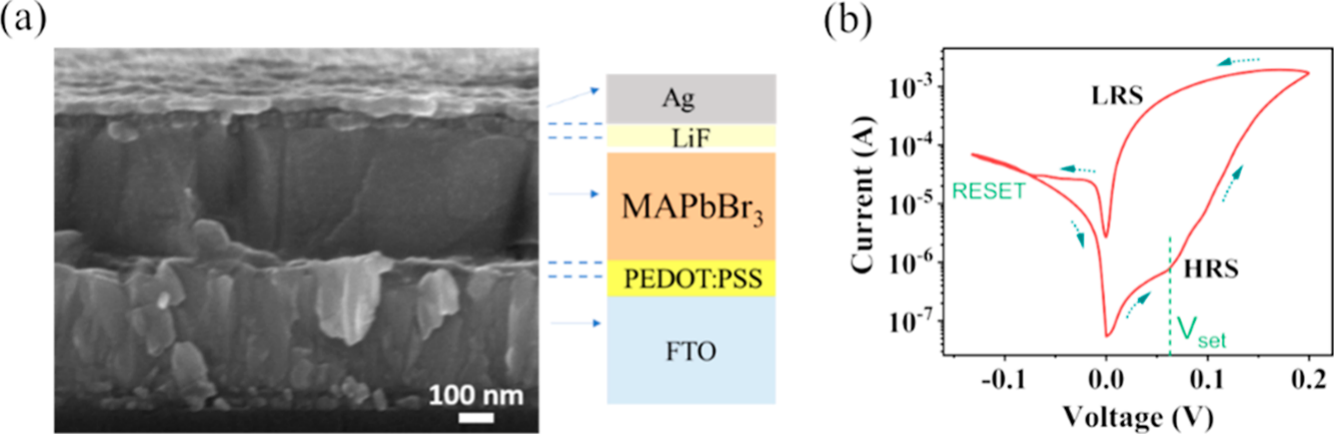
(a) Cross-sectional SEM image of the device alongside a schematic representation of its respective layers. (b) Cyclic voltammogram of the device recorded at a scan rate of 1 mV/s.

To evaluate the current–voltage response of the device, cyclic voltammetry (CV) was recorded at a scan rate of 1 mV/s (see Figure 1b). In the initial stage of the CV curve, at a low voltage (<50 mV), the device exhibited low currents of about 10^–6^–10^–7^ A, indicating its high-resistance state (HRS). Upon reaching a threshold voltage of *V*_set_ = 70 mV, the current increased exponentially by 3 orders of magnitude, transitioning the device to its low-resistance state (LRS). Rather than an abrupt switching, a gradual transition was observed, underscoring the device’s bistable resistive behavior. These gradual transitions are typically associated with the migration of species across the large thickness of the perovskite material.^21^ This characteristic is essential for potential applications in analogous memory and switching devices, where a reliable and repeatable resistance state is indispensable. The response of the cyclic voltammetry is very robust with little current evolution during the initial 1000 CV cycles (Figure S1). The device yield of these memristors with a LiF buffer layer is higher than 90% (Figure S2).

The working mechanism of the devices fabricated in this work involves the formation of conductive filaments, which probably arise from halide vacancies and the migration of lithium ions that migrate through the perovskite layer. Although the conductive filaments originating from halide vacancies have been well studied for perovskite-based memristors, the threshold voltages required to switch from HRS to LRS during the CV measurement are typically high (>1 V). However, these threshold potentials can be significantly reduced by employing preoxidized top metal contacts. In this study, we report threshold potentials below ∼70 mV at low scan rates attributed to Li^+^ migration-driven conductivity changes in the perovskite. Importantly, an abrupt switching between HRS and LRS was not observed, indicating that conductive metal filaments are not responsible for the activation process. Note that the reduction of Li^+^ to Li^0^ is thermodynamically unfavorable and requires a high applied voltage (>3.2 V) to promote this electrochemical reduction. Instead, we attribute the progressive conductivity transition to doping of the perovskite associated with the migration of Li^+^ ions. At low applied negative potentials, the device deactivates, leading to the original HRS; see Supporting Information for further cyclic voltammetry experiments.

The gradual increase in the electrical conductivity emphasizes the suitability of the device for use in analog logic circuits and synaptic device fabrication. Its ability to implement analog switching at low applied potential positions halide perovskites as promising materials for the reproducible and energy-efficient implementation of electronic and neuromorphic systems. To evaluate its performance, low-voltage training pulses (450 mV for 5 ms duration) with defined patterns were applied, as shown in Figure 2a–c. For example, voltage pulses with the sequence pulse-null-null-pulse-pulse–pulse (null = 0 mV during 5 ms) were applied, and the device accurately reflected the input pattern at a read current of 450 mV, demonstrating its reliable reading characteristics. It is worth noting that increasing the number of consecutive pulses results in a gradual increase in the reading current. These results highlight the possibility of implementing analog switching by increasing the number of consecutive pulses, which is suitable for applications such as logic circuits and neuromorphic systems. Further complementary measurements are shown in the Supporting Information to test various conditions (Figures S3 and S4).

**Figure 2:**
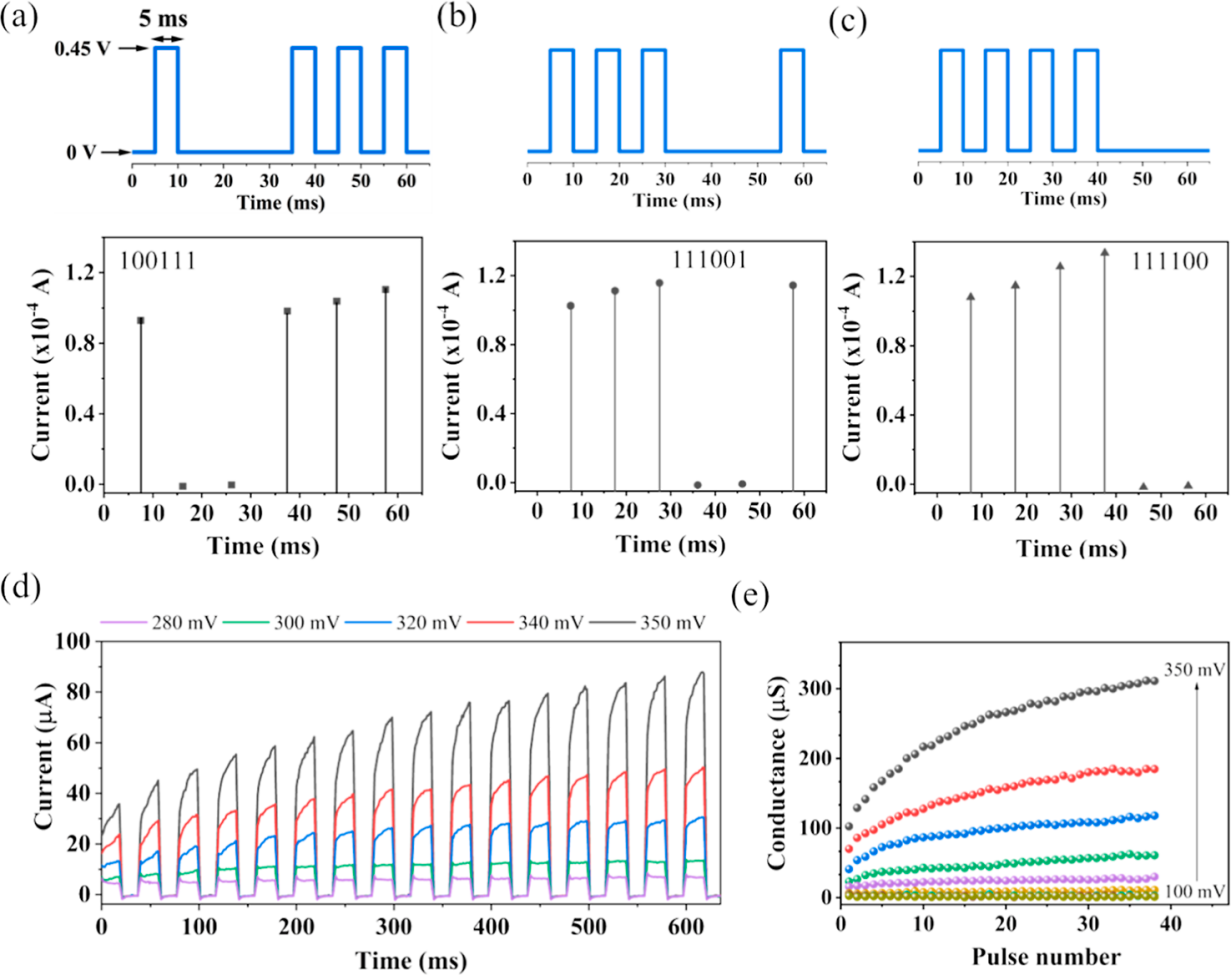
(a–c) Demonstration of device coding via analog switching, illustrating binary states: 100111 (a), 111001 (b), and 111100 (c). (d) Linear potentiation curves of the devices under different impulse sequences (pulse width: 20 ms; interval: 15 ms; base voltage: 0 V; pulse voltage: 280–350 mV). (e) Memristor conductance trends extracted from (d).

To gain a deeper understanding of the dynamic switching response of the device, long trains of voltage pulses were sequentially applied from low voltages of 280 mV to 350 mV (Figure 2d). At low voltages using a 20 ms pulse, the device does not potentiate, and when voltages approach 300 mV, the device begins to potentiate. The increase in conductance observed during the first pulse, compared to the lower SET potentials of the last few cycles, highlights the rise in the SET activation process. Due to the small size of Li^+^ ions, a small increase in the voltage of 20 mV leads to significant differences in the potentiation curves in the range of 300–350 mV. This underscores the exceptional sensitivity of the device with energy consumption in the range of previously reported values. Here, as a first approximation, the energy consumption can be calculated as ∼30 nJ, using *E* = *I* × *V* × *t* for a pulse of 300 mV, *t* = 20 ms, and *I* = 5 μA. To further analyze its conductive behavior, we extracted conductivity-level information from Figure 2d using the current of the last measurement of each pulse cycle and the applied SET potential. This process allowed us to determine the peak conductivity positions, as collected in Figure 2d. The resulting data shown in Figure 2e demonstrate a linear relationship, with conductivity levels reaching several 10^–6^ S in correspondence with their respective SET applied potentials. Thus, the resulting curve illustrates the plasticity of the conductivity and shows its dependence on the applied potential difference of 20 mV and the number of pulses. The energy consumption and conductivity levels match previous results reported for halide perovskite materials, but in our work, the system is very sensitive to applied voltage variations. Furthermore, the device’s sensitivity to the voltage is demonstrated by a multilevel endurance measurement. Further information is shown in Figure S5a, where a sequence of 28 consecutive pulses with an amplitude of 500 mV is applied to evaluate the device’s response. Following this, a RESET voltage of −50 mV for 15 s is applied to transition the device. The results indicate that the device effectively responds linearly to the applied voltage pulses and subsequently transitions to a highly resistive state upon RESET operation. The color code represents the device’s response to the applied potential and its corresponding order. Although the device’s conductance was measured in the range of a few microsiemens, slight variations were observed in different cycles. After the reset process, the conductivity decreased slightly due to the increase in the capacitive resistance at the interfaces. This behavior indicates that Li^+^ ions are attracted to the negative potential to form a double layer. However, after applying a few SET activation pulses, a linear increase in the device’s conductance was observed, demonstrating its capacity for consistent performance under successive operations. Simultaneously, when the SET potential is increased to 500 mV for a duration of 500 ms, the conductivity rises to the milli-Siemens range, as shown in Figure S5. During retention experiments (Figure S9), it was observed that the increase of conductance lasts for more than 1 h by applying a low voltage (0.5 V) pulse. Regarding the stability, devices retain the memory response when stored in the absence of moisture, as shown in Figure S10. Similarly, if devices are protected from moisture with a thin layer of PMMA, they also work adequately after 120 days of storage in the dark under ambient conditions.

In order to gain a better understanding of the stability of the device during writing (potentiation) and erasing (depression) cycles, experiments, as shown in Figure 3, were conducted. Figure 3a shows the potentiation and depression as a function of the applied pulse voltage from 130 to 400 mV for preconditioned devices. A series of 20 consecutive voltage pulses (each pulse lasting 30 ms) was applied to write/delete the information to the device to test the linearity of the writing/deleting process. The device demonstrates a distinct and clearly observable response at applied potentials as low as 130 mV. Notably, the reset potential was maintained at −15 mV, regardless of the writing potentials applied, and was consistent with the number of pulses required to erase the memory. Throughout the writing and erasing processes, the conductivity level of the device was consistently recorded using a read voltage of 30 mV, with each measurement lasting 30 ms. As can be observed, conditions can be found to be relatively linear for potentiation measurements, specifically at low applied voltages (V ∼ 130–180 mV). Alternatively, depression is highly linear under most conditions. It is important to note that this data was obtained after the so-called electroforming, which in practical terms is used to initially distribute the ions through the bulk of the halide perovskite. This conditioning treatment was carried out by applying a 450 mV pulse during 5 s. For this reason, the device is able to activate at lower voltages than those in Figure 2. Furthermore, as shown in Figure 3b, endurance testing reveals suitable performance, with the device successfully enduring 10,000 cycles at 180 mV. The corresponding pulse structure used for these operations is depicted in Figure 3c. These results highlight the efficiency of the LiF buffer layer in achieving a highly stable and robust device, making it a promising candidate for the development of memory systems tailored for neuromorphic applications.

**Figure 3:**
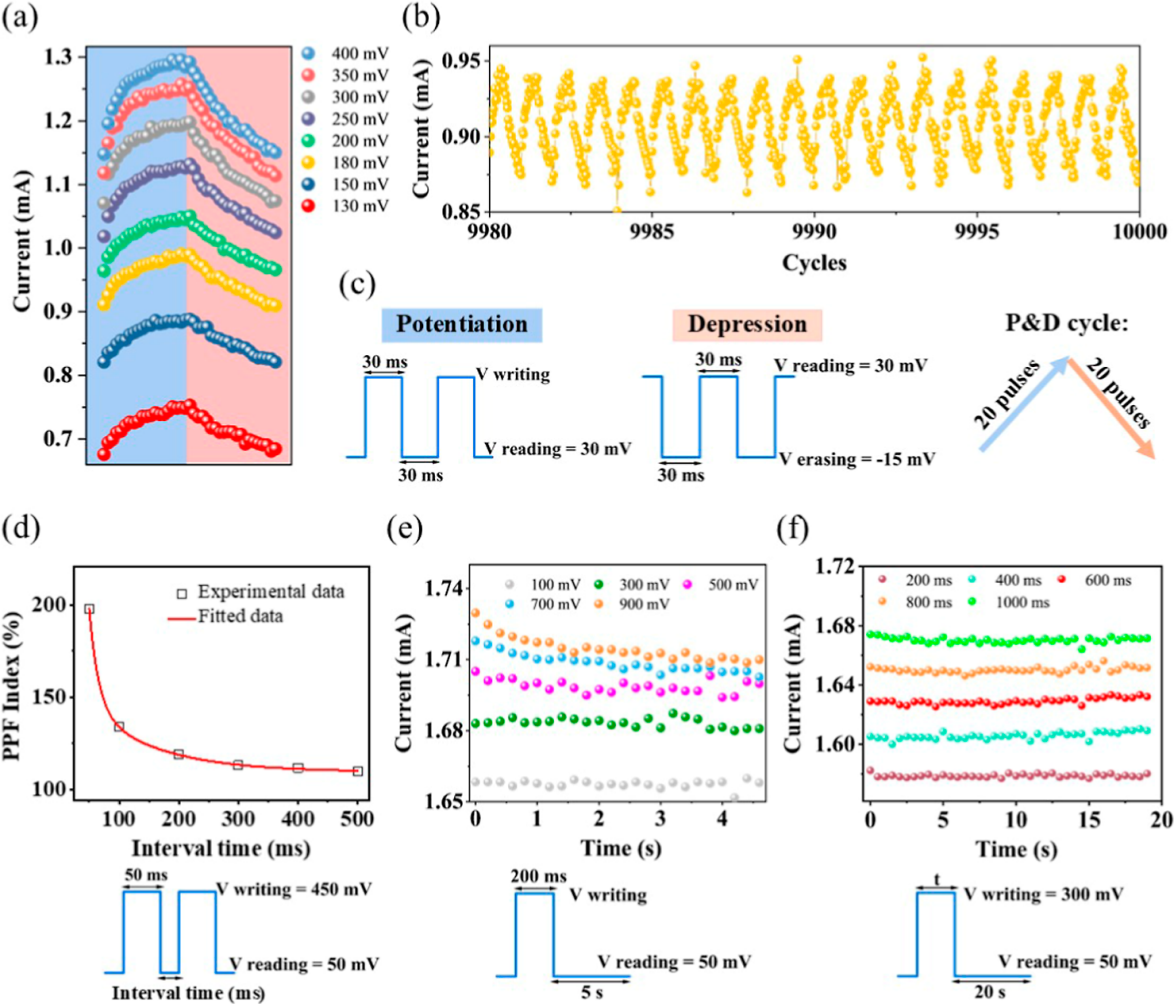
Study of the synaptic plasticity of the memristor. (a) Potentiation and depression (P&D) cycles of the device under writing voltages from 130 to 400 mV. (b) P&D cycle stability at a writing voltage of 180 mV over 10,000 cycles. (c) Pulse voltage–time program applied to obtain the P&D cycles. (d) Paired-pulse facilitation (PPF) index of the device. (e) Spike voltage-dependent plasticity of the device. (f) Spike-width-dependent plasticity of the device. The pulse–time program applied for (c), (e), and (f) is described below each figure.

To further explore the role of the LiF buffer layer, we fabricated reference devices for comparison. All of the devices were compared upon applying the same pulse program shown in Figure 3c, using low *V*_writing_ values of 150, 180, and 200 mV. On the one hand, we fabricated devices without the LiF buffer layer (Ag top electrode in contact with the perovskite layer). While the LiF-modified device exhibited suitable potentiation/depression characteristics (symmetric, linear, and significant current change) at all of the *V*_writing_ tested values, the devices without LiF showed no potentiation/depression response at any voltage tested (Figure S6). This result evidence the role of the LiF buffer layer in decreasing the threshold voltage at which the resistive switching occurs. On the other hand, to verify the role of the Li^+^ from the buffer layer in the synaptic behavior, we also prepared devices replacing the LiF with AgF. The device with the AgF buffer layer also showed no potentiation/depression response at *V*_writing_ = 150 and 180 mV (Figure S6), supporting the role of Li^+^ in the synaptic behavior. This comparison also rules out the possibility that the resistive switching at lower set voltages is caused by the formation of an AgF interfacial layer between the LiF and the Ag top electrode. At *V*_writing_ = 200 mV, the devices with the AgF buffer layer start to show linear potentiation behavior, but the change in conductance is lower than in the LiF devices. This agrees with our previous works, where we proved that the addition of a buffer layer with preoxidized Ag (e.g., AgI) decreases the SET voltage for the resistive switching in glass|FTO|PEDOT/PSS|MAPbBr_3_|Ag memristors.^19,20^

Synaptic plasticity measurements are essential for assessing the suitability of a device for neuromorphic computations. In this study, we measured paired-pulse facilitation (PPF) using the pulse structure illustrated in Figure 3d (pulse width of 50 ms). The interval between the paired pulses (Pulse 1 and Pulse 2) varied from 50 to 500 ms, and the device’s response was recorded. The ratio of the response to Pulse 2 relative to Pulse 1 initially showed a significant weight gain of about 200%. Within an interval of 100 ms, this enhancement gradually decreased to 100% and subsequently remained stable, demonstrating the device’s volatile behavior over extended intervals. To further investigate the plasticity of the device, the excitatory postsynaptic currents (EPSCs) were recorded in Figure 3e at a potential of 50 mV after applying a single voltage pulse (spike) with a duration of 200 ms. The results demonstrated that the amplitude of the EPSC gradually increased with an increase in the spike potential from 100 to 900 mV. Additionally, when the spike potential was kept constant at 300 mV and the spike duration was varied from 200 to 1000 ms, the device exhibited a stable conductive state, with the amplitude of the EPSC gradually increasing with the longer spike duration (see Figure 3f). These findings highlight the device’s robust plasticity and its ability to respond to variations in spike potential and duration, underscoring its potential to mimic synaptic behavior.

To demonstrate the concept of this device for neuromorphic computing applications, we used this device in a deep neural network (DNN) to classify handwritten digits from the MNIST data set.^22^ The long-term plasticity (potentiation/depression) of memory observed in the experiments is mapped to represent the adjustable synaptic weights in this network. Figure 4a presents the evolution of recognition accuracy over 50 epochs during training, investigating the effect of changes in the spike potential from 150 to 400 mV. The simulation results show successful learning with the accuracy increasing rapidly and reaching saturation at all tested voltages. After 50 training sessions, the recognition accuracy reached 89% at spike potential voltages of 150–180 mV. Although the accuracy decreased slightly after this peak, potentially indicating the onset of mild overfitting or suggesting an optimal training duration, the results clearly demonstrate effective learning facilitated by the operation of memristor synapses at low voltages. Furthermore, we used the MNIST test set to evaluate the network’s ability to classify data that it has not seen before. Figure 4b shows the confusion matrix performed under the optimal 180 mV condition. The main diagonal confirms that the DNN using the halide perovskite memristors accurately classifies unseen digits using low writing voltages and thus lower power consumption. Variation of the DNN model can provide a more sensitive response to the voltage pulses, as shown in the Supporting Information. Overall, these findings provide strong evidence for the potential of the glass|FTO|PEDOT/PSS|MAPbBr_3_|LiF|Ag device for neuromorphic computing.

**Figure 4:**
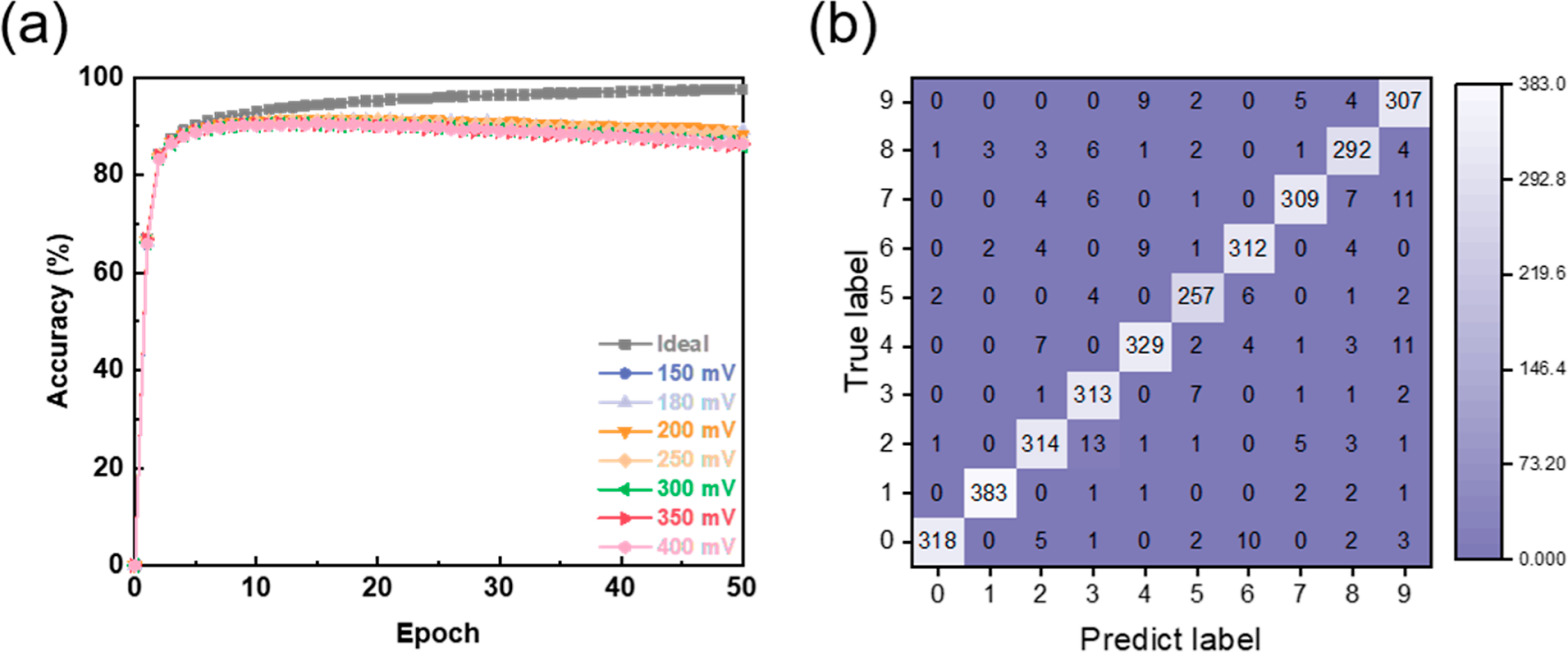
(a) Recognition accuracy of the device with different spike potentials. (b) Confusion matrix for a handwritten digit classification-based device with 180 mV as potentials.

## Conclusion

In summary, an interlayer of LiF is used as a source of Li^+^ ions that facilitate the formation of conductive filaments across the perovskite layer at a low applied voltage. The device demonstrated excellent performance, leading to linear potentiation using a train of voltage pulses enabling information to be written at a remarkably low voltage of 130 mV and erased at an equally low negative potential of −15 mV. Moreover, the device exhibited stable and reliable writing–erasing cycling stability, exceeding 10^4^ cycles, and demonstrated multilevel endurance and a coding response with the added benefit of analog switching. The observed linear potentiation curves allow the precise control of the device conductivities to the level of several microsiemens that allows their use in deep neural networks using low writing voltages with acceptable results. This work demonstrates the importance of interfacial buffer layers in halide perovskite memristors, which will help to enhance their future applications. Importantly, the use of LiF as an interlayer should be universally valid for other families of materials used in memristor applications.

## Experimental Section

### Materials

Lead bromide (PbBr_2_, TCI, 98%), methylammonium bromide (MABr, Greatcell, 99.99%), lithium fluoride (LiF, Sigma-Aldrich, 99.98%), poly­(3,4-ethylenedioxythiophene) polystyrenesulfonate (PEDOT/PSS, Heraeus), FTO glass substrates (Pilkington, 15 Ω/cm^2^), zinc (Zn dust, size <10 μm, >98%), hydrochloric acid (HCl, 37%, Sigma-Aldrich), and ultrapure water (Millipore) are used. Solvents such as *N*,*N*-dimethylformamide (DMF, anhydrous, 99.8%), dimethyl sulfoxide (DMSO, anhydrous, 99.9%), and toluene (anhydrous, 99.8%) are procured from Sigma-Aldrich; other solvents such as acetone (>99%) and isopropyl alcohol (>99%) are procured from VWR CHEMICALS, France.

### Fabrication Methods

Prior to the device fabrication, the conductive (F/SnO_2_) FTO substrates are chemically etched using Zn powder and hydrochloric acid (HCl) to obtain the required conductive patterns. These substrates are then cleaned using the detergent, acetone, and isopropyl alcohol sequentially by placing them inside an ultrasonic bath, each for 10 min, respectively. Then, after purging these substrates with dry air, they are placed inside an ultraviolet (UV) chamber for 15 min to further clean the FTO surface. Without further delay, over these substrates, the commercial PEDOT/PSS solution (filtered using a syringe filter (Ø = 450 nm)) is spin-coated (the spin speed is set to 3000 rpm during 30 s). These glass|FTO|PEDOT/PSS substrates are heated at 120 °C for 5 min in an open air condition, followed by 10 min in an inert condition.

### MAPbBr_3_ Deposition

Inside a nitrogen-filled glovebox, PbBr_2_ (513.8 mg) is taken into a vial. To the vial with PbBr_2_, 800 μL of DMF and 200 μL of DMSO are added, and after 2 h, the PbBr_2_ is found to be dissolved completely, and then MABr (156.7 mg) is added to it to prepare the perovskite precursor solution. The perovskite films were grown on top of glass|FTO|PEDOT/PSS substrates by spin-coating inside the glovebox, and the oxygen and moisture levels are maintained to be less than 0.1 ppm. During the perovskite deposition (5000 rpm and 40 s), 10 s before stopping the spin, toluene (400 μL) is dropped over the substrate to act as an antisolvent. Without further delay, the substrates were heated over a hot plate at 120 °C for 30 min. The substrate glass|FTO|PEDOT/PSS|MAPbBr_3_ remained inside the glovebox until we deposited the buffer layer and top metal contacts.

### LiF and Silver Deposition

The other two layers are deposited over the glass|FTO|PEDOT/PSS|MAPbBr_3_ substrates by thermal evaporation. Pellets of LiF are prepared by using a cold press, introducing LiF powder in a die, and applying an axial pressure of 5 tons. LiF and Ag are evaporated in the vacuum at a base pressure of 8.5 × 10^–6^ Torr. The evaporation of LiF and Ag is carried out with great control of the evaporation rate. Initially, a 30 nm thick LiF is evaporated (0–1 nm thick LiF: 0.5 Ås^–1^, 1–10 nm thick LiF: 0.5 Ås^–1^, and 10–30 nm thick LiF: 5 Ås^1–^), and then a 50 nm Ag is deposited (0–1 nm thick Ag: 0.5 Ås^–1^, 1–10 nm thick Ag: 1 Ås^–1^ and 10–50 nm thick Ag: 5 Ås^–1^).

### Electrical Measurements

All electrical measurements were carried out inside the glovebox using a Metrohm’s Autolab PGSTAT204 coupled with Nova 2.1 software with the help of a sample holder.

### Neural Network Computing

The deep neural network (DNN) architecture consists of an input layer with 784 neurons (corresponding to 28 × 28 digit data), two hidden layers with 256 and 64 neurons, respectively, and an output layer with 10 neurons. These 10 output neurons correspond to the classification of digits (0–9). All DNN computations were performed using a Python-coded program. The DNN model employs synaptic weight based on the conductance changes in perovskite memristors.

## Supplementary Material



## Notes

Memristor device fabrication, stabilized characteristic I–V switching response, redox reaction of the MAPbI_3_/Ag/Au device, and dynamical model fitting resources (PDF)

## References

[1] Ng S., John R. A., Yang J. T., Mathews N. (2020). Forming-Less Compliance-Free
Multistate Memristors as Synaptic Connections for Brain-Inspired Computing. ACS Appl. Electron. Mater..

[2] Park H. L., Lee T. W. (2021). Organic and Perovskite Memristors for Neuromorphic
Computing. Org. Electron..

[3] Van
De Burgt Y., Lubberman E., Fuller E. J., Keene S. T., Faria G. C., Agarwal S., Marinella M. J., Alec Talin A., Salleo A. (2017). A Non-Volatile Organic Electrochemical
Device as a Low-Voltage Artificial Synapse for Neuromorphic Computing. Nat. Mater..

[4] Seo K., Kim I., Jung S., Jo M., Park S., Park J., Shin J., Biju K. P., Kong J., Lee K., Lee B., Hwang H. (2011). Analog Memory and Spike-Timing-Dependent Plasticity
Characteristics of a Nanoscale Titanium Oxide Bilayer Resistive Switching
Device. Nanotechnology.

[5] Yang J. J., Pickett M. D., Li X., Ohlberg D. A. A., Stewart D. R., Williams R. S. (2008). Memristive Switching
Mechanism for Metal/Oxide/Metal
Nanodevices. Nat. Nanotechnol..

[6] Lee J., Yang K., Kwon J. Y., Kim J. E., Han D. I., Lee D. H., Yoon J. H., Park M. H. (2023). Role of Oxygen Vacancies
in Ferroelectric or Resistive Switching Hafnium Oxide. Nano Convergence.

[7] Kang K., Hu W., Tang X. (2021). Halide Perovskites
for Resistive Switching Memory. J. Phys. Chem.
Lett..

[8] Xiao X., Hu J., Tang S., Yan K., Gao B., Chen H., Zou D. (2020). Recent Advances in
Halide Perovskite Memristors: Materials, Structures,
Mechanisms, and Applications. Adv. Mater. Technol..

[9] Gogoi H. J., Bajpai K., Mallajosyula A. T., Solanki A. (2021). Advances in Flexible
Memristors with Hybrid Perovskites. J. Phys.
Chem. Lett..

[10] Stathopoulos S., Khiat A., Trapatseli M., Cortese S., Serb A., Valov I., Prodromakis T. (2017). Multibit Memory
Operation of Metal-Oxide
Bi-Layer Memristors. Sci. Rep..

[11] Bisquert, J. ; Garcia-Belmonte, G. ; Guerrero, A. Ionic/Electronic Conduction and Capacitance of Halide Perovskite Materials. In Perovskite Photovoltaics and Optoelectronics: From Fundamentals to Advanced Applications; Wiley, 2022; pp 173–213. 10.1002/9783527826391.ch6.

[12] Tress W. (2017). Metal Halide
Perovskites as Mixed Electronic-Ionic Conductors: Challenges and Opportunities
- From Hysteresis to Memristivity. J. Phys.
Chem. Lett..

[13] Kim H., Yang S. J., Shim Y. S., Moon C. W. (2025). A Comprehensive
Review of Electrochemical Metallization and Valence Change Mechanisms
in Filamentary Resistive Switching of Halide Perovskite-Based Memory
Devices. ACS Appl. Mater. Interfaces.

[14] Sakhatskyi K., John R. A., Guerrero A., Tsarev S., Sabisch S., Das T., Matt G. J., Yakunin S., Cherniukh I., Kotyrba M., Berezovska Y., Bodnarchuk M. I., Chakraborty S., Bisquert J., Kovalenko M. V. (2022). Assessing
the Drawbacks and Benefits of Ion Migration in Lead Halide Perovskites. ACS Energy Lett..

[15] Gonzales C., Guerrero A. (2023). Mechanistic and Kinetic Analysis of Perovskite Memristors
with Buffer Layers: The Case of a Two-Step Set Process. J. Phys. Chem. Lett..

[16] Xiao X., Hu J., Tang S., Yan K., Gao B., Chen H., Zou D. (2020). Recent Advances in Halide Perovskite
Memristors: Materials, Structures,
Mechanisms, and Applications. Adv. Mater. Technol..

[17] Wu Y., Wei Y., Huang Y., Cao F., Yu D., Li X., Zeng H. (2017). Capping CsPbBr3 with ZnO to Improve Performance and Stability of
Perovskite Memristors. Nano Res..

[18] Hwang B., Lee J. S. (2017). Hybrid Organic-Inorganic
Perovskite Memory with Long-Term
Stability in Air. Sci. Rep..

[19] Pérez-Martínez J. C., Berruet M., Gonzales C., Salehpour S., Bahari A., Arredondo B., Guerrero A. (2023). Role of Metal Contacts
on Halide Perovskite Memristors. Adv. Funct.
Mater..

[20] Pendyala N., Gonzales C., Guerrero A. (2025). Decoupling Volatile
and Nonvolatile
Response in Reliable Halide Perovskite Memristors. Small Struct.

[21] Gonzales C., Guerrero A., Bisquert J. (2021). Spectral Properties
of the Dynamic
State Transition in Metal Halide Perovskite-Based Memristor Exhibiting
Negative Capacitance. Appl. Phys. Lett..

[22] Sanjuán I., Franco D., Chen Q. G., Chueh C. C., Lee W. Y., Guerrero A. (2025). Proton Migration-Modulated n-Doped Poly­(benzodifurandione)
Organic Electrochemical Transistors Used for Neuromorphic Computing
Applications. ACS Energy Lett..

