# Supplementary material for: A Lithium Fluoride Interfacial Layer for Low-Voltage and Reliable Perovskite Memristors
Source: ACS Appl Electron Mater. 2025 Dec 19;8(1):645–51. doi: 10.1021/acsaelm.5c02347 (PMC12805799; doi:10.1021/acsaelm.5c02347)
Supplement: Supplementary file 1 [file el5c02347_si_001.pdf]

Supporting Information

Lithium Fluoride Interfacial Layer for Low Voltage and Reliable Perovskite Memristors

Naresh Kumar Pendyala,[1] Ignacio Sanjuán,[1] Qun-Gao Chen,[2] Wen-Ya Lee,[2] Chu-Chen Chueh,[3] and Antonio Guerrero [1]*

[1]Institute of Advanced Materials (INAM), Universitat Jaume I, 12006 Castelló, Spain.
[2]Department of Chemical Engineering and Biotechnology, National Taipei University of Technology, Taipei 106344, Taiwan
[3]Department of Chemical Engineering, National Taiwan University, Taipei 10617, Taiwan

Corresponding authors: A. Guerrero (aguerrer@uji.es)

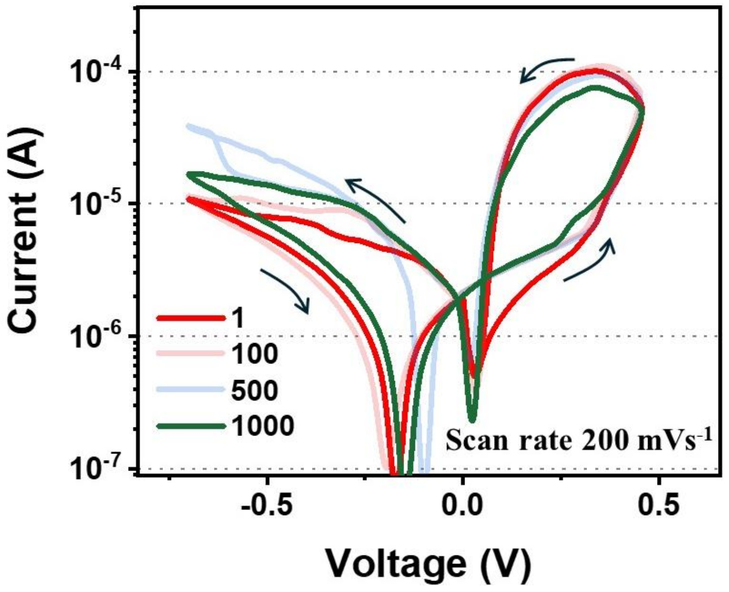

Figure S1: Stability of the device during cyclic voltammetry measurements conducted at a scan rate of 200 mV s^{-1}.

I-V curves shown in Figure S1 do not pass through the origin because of the scan rate used, 200 mV/s. This is due to the capacitive behavior of the memristor. Higher scan rates lead to higher contributions of the capacitive current at voltages close to the origin. This capacitive behavior of perovskite-based memristor devices is analysed in our previous publication, Pendyala et al. (2025).[1]

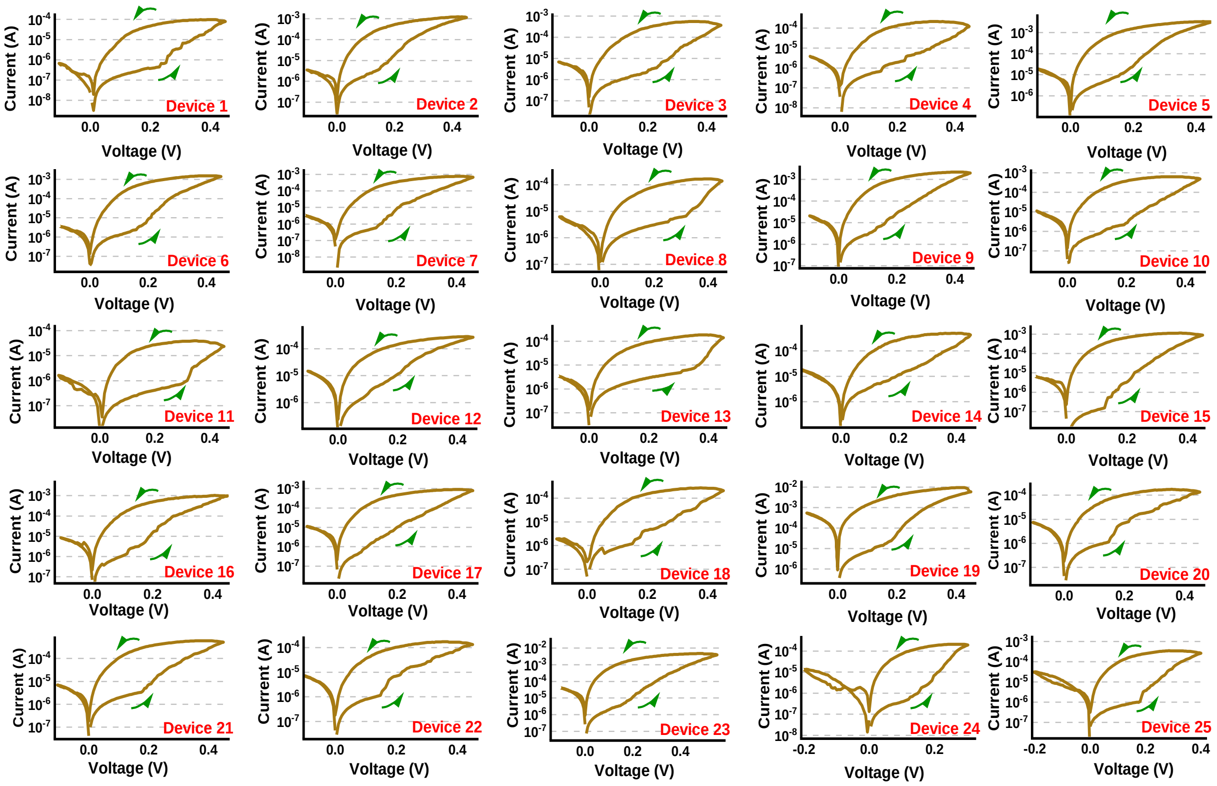

Figure S2. Reproducibility in device fabrication. I-V curves registered for 25 different memristors with the LiF buffer layer from the same batch. The analysis of the I-V curves reveals a device yield of >90%.

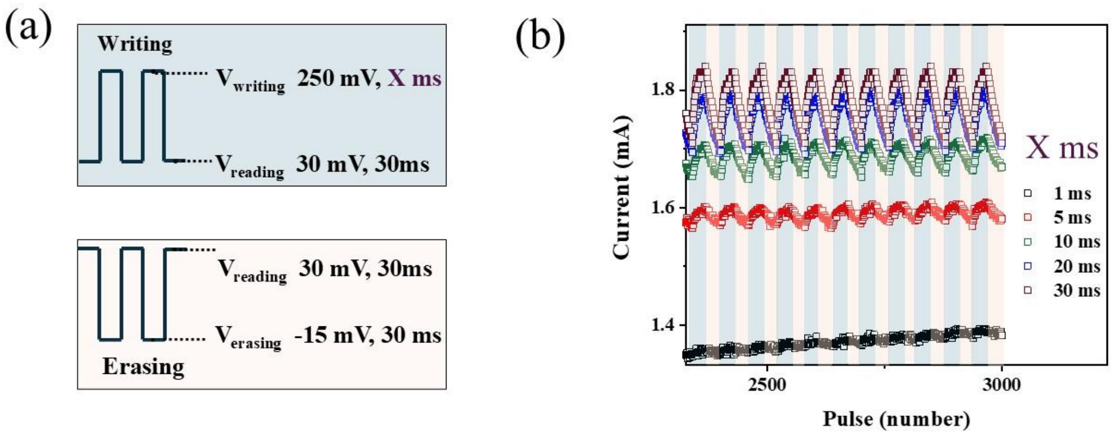

Figure S3: The writing-erasing characteristics of the device were investigated using the specified pulse structure (a). The writing-erasing cycles of the device at various writing durations ranging from 1 to 30 ms are shown in (b).

Complete set of measurements obtained for Figure 2 shown in the main manuscript.

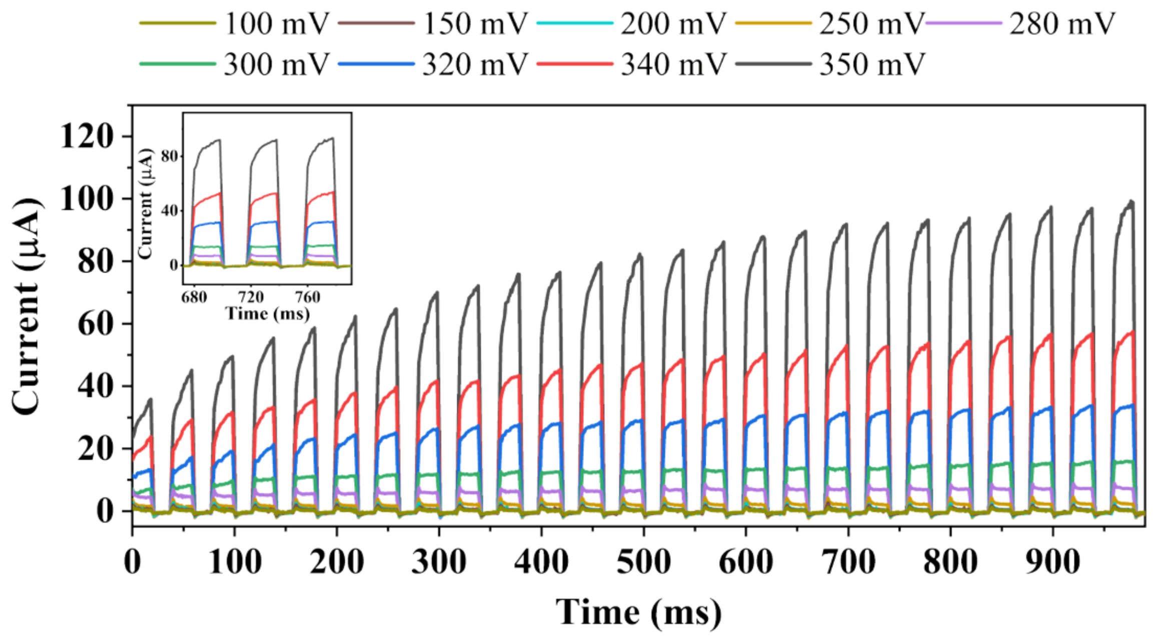

Figure S4: Linear potentiation curves of the devices under different impulse sequences (pulse width: 20 ms; interval: 15 ms; base voltage: 0 V; pulse voltage: 100–350 mV).

Each vertical line observed in Fig. S4 correspond to a complete cycle of 28 pulses, the next vertical line is obtained after a reset process.

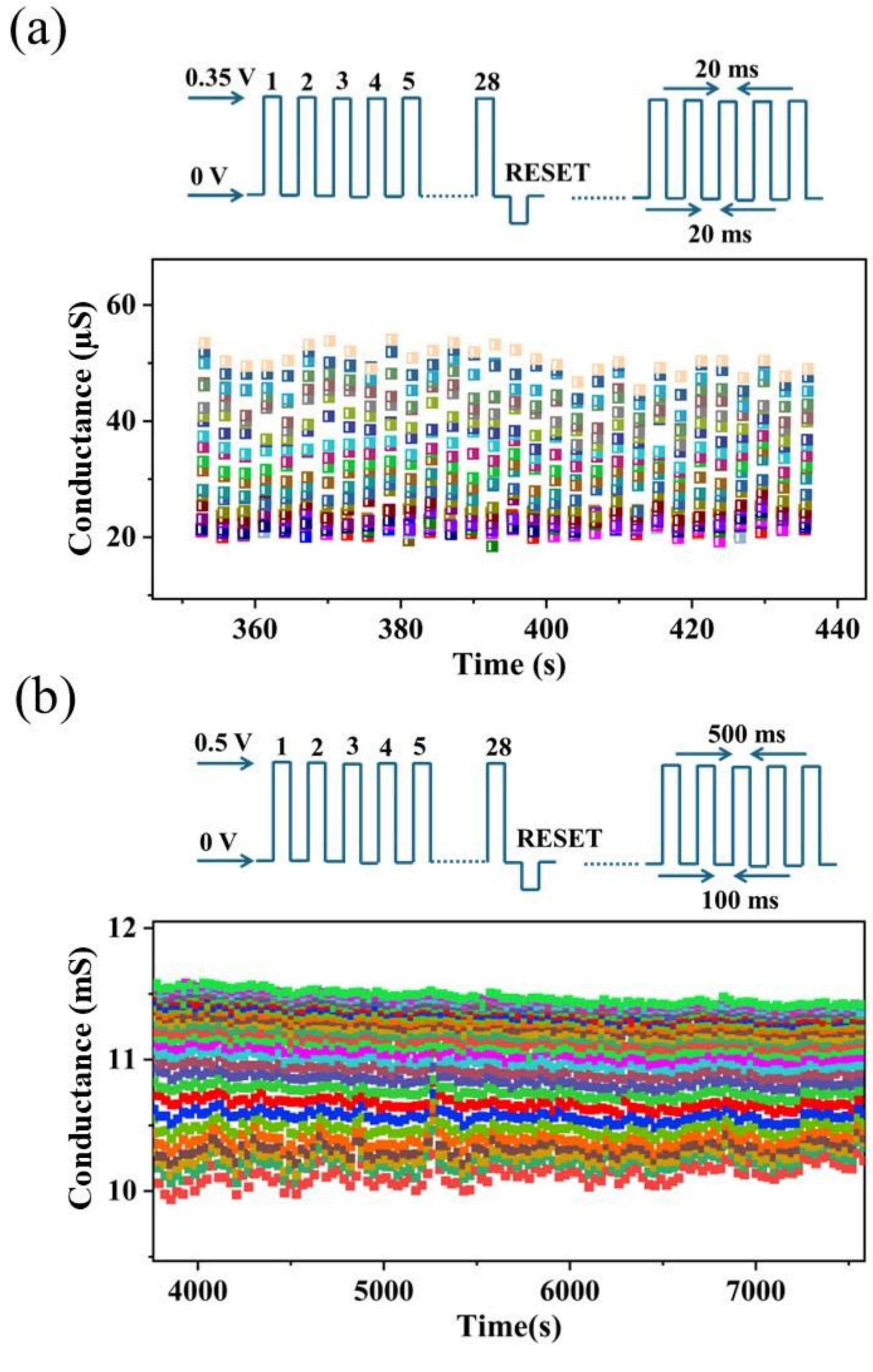

Figure S5: The multilevel endurance of the device, showing repeated cycles with 28 continuous impulses followed by a reset. a) Voltage amplitude: 350 mV, pulse width: 20 ms, interval time: 20 ms, base voltage: 0 V. b) Voltage amplitude: 500 mV, pulse width: 500 ms, interval time: 100 ms, base voltage: 0 V.

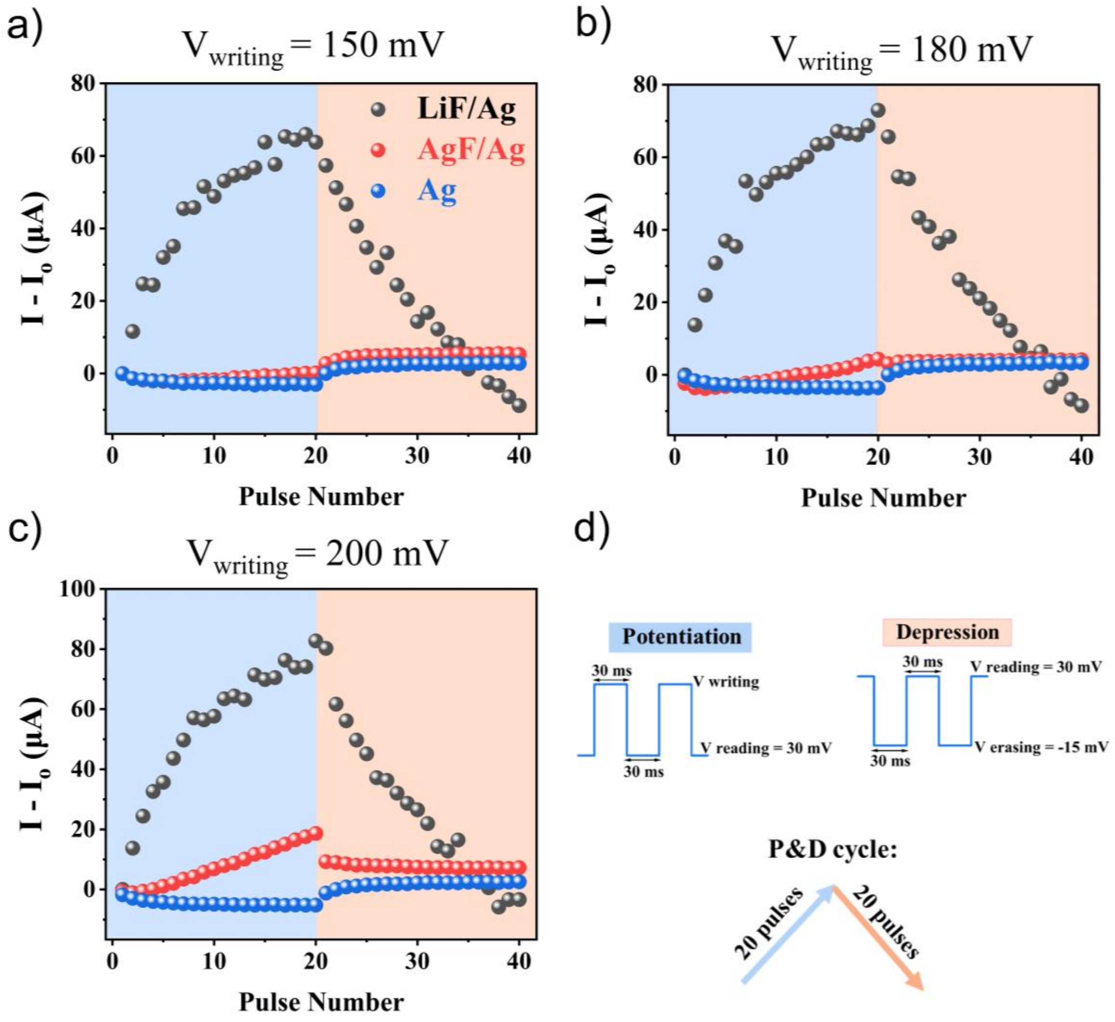

Figure S6: Comparison of the LTP/LTD curves obtained for memristors with different configuration ITO/PEDOT:PSS/$MAPbBr_3$/**BF**/Ag (**BF**: LiF, AgF, or BF-free). a) Writing voltage = 150 mV, b) writing voltage = 180 mV, and c) writing voltage = 200 mV. d) Description of the pulse voltage program applied to construct the LTP/LTD curves.

Table S1: Summary of the DNN Accuracy for Different Set Potentials

Setting Potentials (mV)	Recognition Accuracy at 50th epoch (%)
150	78.90
180	82.31
200	83.65
250	79.44
300	81.02
350	88.33
400	80.34

Additional DNN models

Modification of the model of the DNN leads to a more sensitive response to the voltage of the applied pulses.

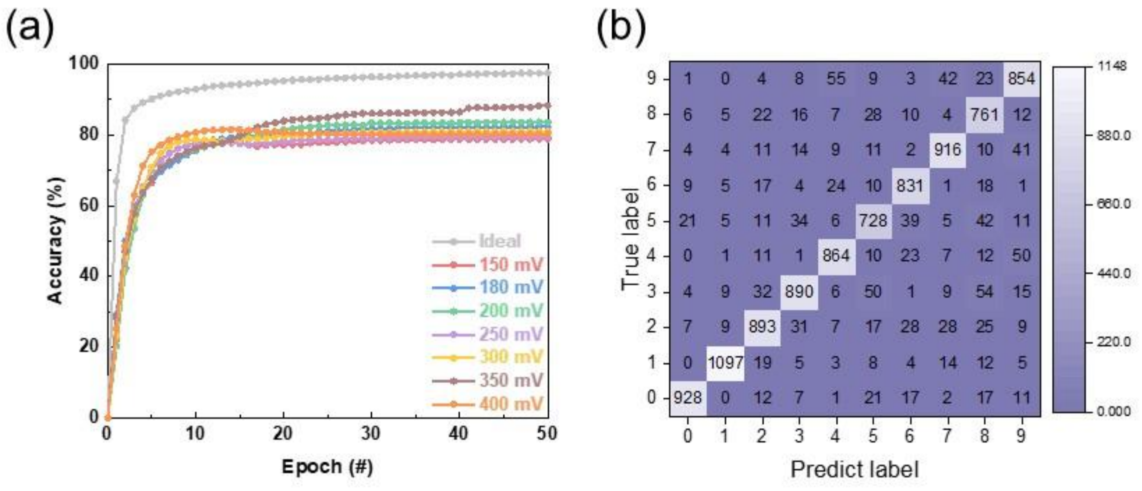

Figure S7: (a) Recognition accuracy of the device with different set potentials. (b) Confusion matrix for handwritten digit classification-based device with 350 mV as potentials.

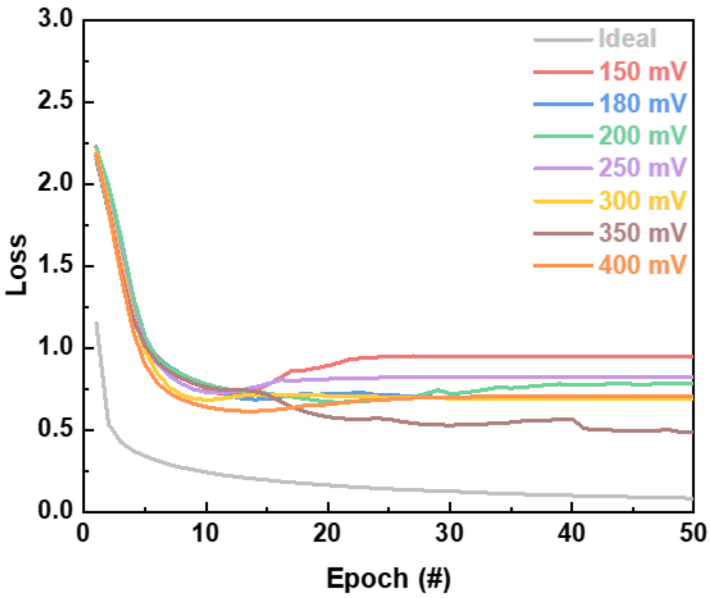

Figure S8: Loss plot of the device with different set potentials ranging from 150 to 400 mV.

Retention time measurement: During the measurement, after the read (100 mV, 1 s), set (500 mV, 1 s), and read (100 mV, 5 s) steps, the device is disconnected from the potentiostat for approximately 6650 s. After this period, the device currents are recorded at a read voltage of 30 mV. The measured currents are found to be higher than the initial values and remain stable, indicating long retention times for the device with the FTO/PEDOT:PSS/$MAPbBr_3$/LiF/Ag configuration.

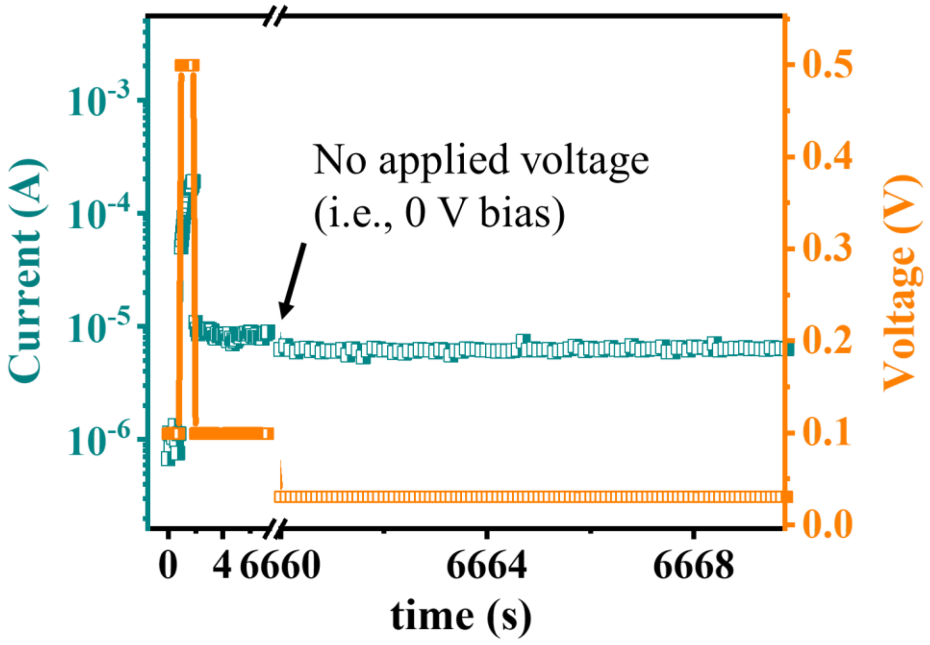

Figure S9: The retention time measurement for the device with the FTO/PEDOT:PSS/$MAPbBr_3$/LiF/Ag configuration.

Device stability test: To evaluate the device stability, we recorded the CV (30 mVs^{-1}) of the freshly fabricated device and then recorded the CV again after 90 days. This measurement demonstrates that the device remains stable after three months of storage inside a nitrogen-filled glovebox.

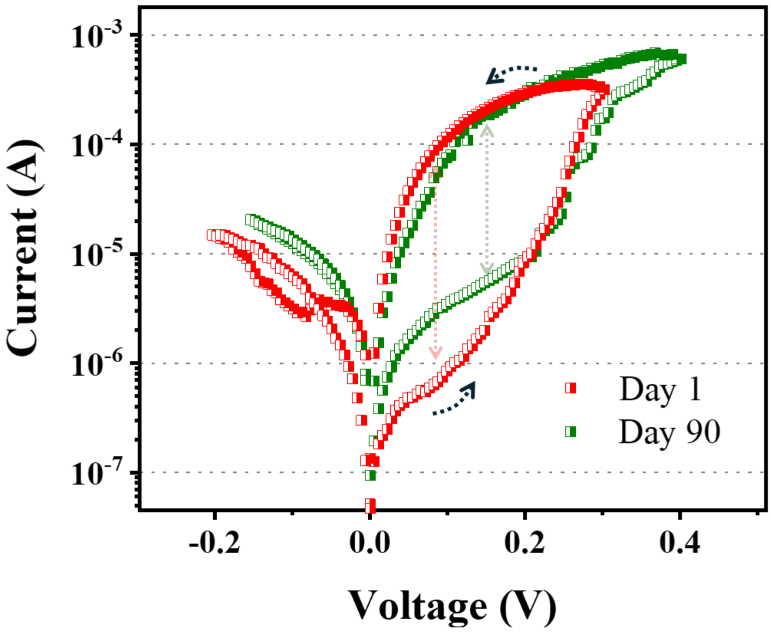

Figure S10: The device stability measurement for the device with the FTO/PEDOT:PSS/$MAPbBr_3$/LiF/Ag configuration.

References

(1) Pendyala, N.; Gonzales, C.; Guerrero, A. Decoupling Volatile and Nonvolatile Response in Reliable Halide Perovskite Memristors. *Small Struct.* **2025**, *6* (1), 2400380. https://doi.org/10.1002/sstr.202400380.
